# High fat diet alters *Drosophila melanogaster* sexual behavior and traits: decreased attractiveness and changes in pheromone profiles

**DOI:** 10.1038/s41598-018-23662-2

**Published:** 2018-03-29

**Authors:** Janna N. Schultzhaus, Chloe J. Bennett, Hina Iftikhar, Joanne Y. Yew, Jason Mallett, Ginger E. Carney

**Affiliations:** 1 Department of Biology, Texas A&M University, College Station, Texas USA; 2 Pacific Biosciences Research Center, University of Hawai’i at Mānoa, Honolulu, Hawai‘i USA; 3 Present Address: Center for Biomolecular Science and Engineering, U.S. Naval Research Laboratory, Washington, D.C. USA; 4 Present Address: Department of Biological Sciences, University of Idaho, Moscow, Idaho USA

## Abstract

Sexual traits convey information about individual quality to potential mates. Environmental and genetic factors affect sexual trait expression and perception via effects on animal condition and health. High fat diet (HFD) is one environmental factor that adversely affects *Drosophila melanogaster* health, and its effects on animal health are mediated through conserved metabolic signaling pathways. HFD decreases female attractiveness, resulting in reduced male mating behaviors toward HFD females. HFD also affects the ability of males to judge mate attractiveness and likely alters fly condition and sexual traits to impact mating behavior. Here we show that HFD affects both visual (body size) and non-visual (pheromone profiles) sexual traits, which likely contribute to decreased fly attractiveness. We also demonstrate that adult-specific HFD effects on male mate preference can be rescued by changing metabolic signaling. These results demonstrate that HFD alters *Drosophila* sexual cues to reflect concurrent effects on condition and that less severe behavioral defects can be reversed by genetic manipulations that rescue fly health. This work expands on current knowledge of the role that metabolic signaling pathways play in linking animal health, sexual traits, and mating behavior, and provides a robust assay in a genetically tractable system to continue examining these processes.

## Introduction

To produce the best quality or greatest number of offspring, animals evaluate potential mates to identify those in the best condition^1–4^. Condition, which is influenced by environmental and genetic factors, is a term commonly employed to describe the internal physiology or overall quality of an animal^5^ and reflects the animal’s fitness potential^6–8^. Mating decisions depend upon the condition of both the potential partner and the choosing animal. Sexual traits, which are thought to convey information about individual fitness potential, and the ability to accurately evaluate this information, are both condition dependent^9–13^. Individuals can differentiate among potential mates of varying quality during mate searching, but searching requires energy and time and therefore can be costly. The benefit that animals in good condition receive when mating with other good condition mates, such as better or more offspring, may not be recouped by poor condition animals if the costs of securing good condition mates exceeds the possible benefits^14–16^. This tradeoff may result in condition-dependent mate preference, where poor condition animals exhibit decreased preference for good condition mates, contributing to assortative matings between good or poor condition pairs.

To identify good condition mates, animals evaluate their potential partner’s sexual traits, which are responsive to environmental and genetic variability (i.e., sexual traits are condition-dependent)^17^. Insect sexual traits include body size, ornament size (e.g., mandibles and horns), courtship song, and pheromone profiles^18,19^. Dietary influences during development can have particularly strong effects on insect sexual traits because insulin signaling controls growth during this critical period^20–23^. Factors that enhance insulin signaling during development will result in larger adult insects, while the reverse is true for factors that reduce insulin signaling, such as reduced nutrient availability^24,25^ or diets high in fat^26^. *D. melanogaster* females prefer large males^27,28^ that are not nutritionally deprived^29^ and that produce energetic courtship songs^30,31^. *D. melanogaster* males prefer females that are large^25^ and have altered pheromone profiles due to elevated insulin signaling^32^.

In ecological studies, “good condition” individuals are often identified as those with high total body lipid stores^33^, yet lipid overconsumption often causes metabolic diseases^34–36^, leading to impaired states of health and lowered life expectancy. The physiological response to dietary lipids is mediated by highly conserved metabolic pathways, such as insulin/TOR and fat lipase signaling. In *D. melanogaster*, an emerging model for metabolic studies^37–39^, diets that are high in fat lead to increased total body lipids^35^, accumulation of lipids in multiple tissues (including the fat body, gut, and heart^35^), insulin resistance^35^, as well as decreased heart function^35,40^, lifespan^41^, and fecundity^42^. Additionally, females raised on HFD are less attractive to males raised on a control diet, and HFD males exhibit condition-dependent mate preference as they do not discriminate between unattractive HFD females and attractive females raised on a control diet^42^. Therefore, exposure to high levels of dietary fat negatively affects fruit fly health and behavior while simultaneously increasing internal fat content, indicating that individuals with high lipid stores cannot always be labeled as “good condition,” especially when considering the effects of dietary imbalances.

Understanding how dietary lipids affect mate choice will be informative for sexual selection studies, as lipid reserves are important determinants of condition and because animals live in fluctuating, complex environments^43^. We previously noted that rearing *D. melanogaster* on 3% HFD throughout development and adulthood (referred to here as “developmental diet”) altered reproductive behavior by causing decreases in female attractiveness and fecundity and male mate discrimination ability^42^. Males use a variety of sensory cues to judge sexual traits of potential mates, and HFD could influence multiple female traits to cause this decrease in attractiveness. We hypothesize that HFD, via the activity of conserved metabolic signaling pathways, alters one or more of these sexual traits to decrease attractiveness. In the current study, we examined: 1) whether HFD modifies any of three major female sexual cues (body size, behavioral responses to courtship, or pheromones) by performing multiple behavioral tests to examine the effect of HFD on each sexual trait in an independent manner; 2) if increased dosage of fat in HFD decreases male attractiveness and, if so, what male sexual traits are affected; 3) whether HFD affects pheromones by quantifying cuticular hydrocarbons (CHCs; several of which function as aphrodisiacs for the opposite sex); 4) and if the behavioral changes associated with exposure to HFD could be rescued by genetically altering metabolic signaling pathways.

## Results

### Males Discriminate Against High Fat Diet Females in Light and Dark Conditions

Raising flies on 3% HFD decreases female body size (Supplemental Fig. 1 and ref.^26^), and we wanted to determine whether this change alone was enough to decrease attractiveness. We compared the behavior and attractiveness of control flies to that of flies raised on 3% HFD (developmental diet) in light and dark conditions. In the dark, males are unable to see and therefore largely judge females based on pheromone profiles rather than visual cues such as body size, although female behavioral response to male courtship advances could impact male performance and mate assessment.

**Figure 1:**
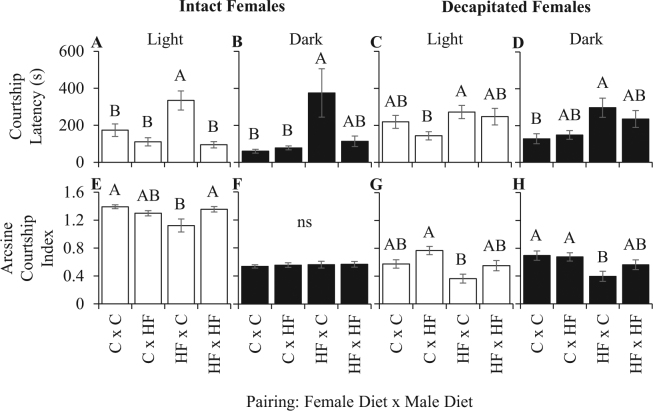
Effects of developmental HFD on behavior. The effect of 3% HFD during development and adulthood on male courtship latency and courtship index towards intact females in light (**A**,**E**) and dark (**B**,**F**) conditions and decapitated females in light (**C**,**G**) and dark (**D**,**H**) conditions. Pairings are female diet X male diet. Each bar represents the mean ± SE of N = 25. The letters above the bars represent a post-hoc Tukey’s HSD where means that do not share the same letter are significantly different at Bonferroni corrected α = 0.01. ns = not significant.

In assays with intact females that were raised on a 3% HFD, male courtship latency was affected in both light and dark conditions (Two-way ANOVA, courtship latency in light: *F*_3,121_* = *12.9183, *P < *0.0001; Fig. 1A; courtship latency in dark: *F*_3,118_* = *5.2346, *P = *0.002; Fig. 1B, Supplemental Table 1), but courtship index was only affected in the light (Two-way ANOVA, courtship index in light: *F*_3,120_* = *4.5272, *P = *0.0048; Fig. 1E; courtship index in dark: *F*_3,117_* = *0.1168, *P = *0.9501; Fig. 1F, Supplemental Table 1). Control males took longer to begin courting intact HFD females in both light and dark conditions but decreased overall courtship towards the HFD females only in the light. Conversely, males fed the 3% HFD courted control and HFD females similarly across all visual conditions.

Light or dark condition assays also were performed with decapitated females. Decapitation removes the female’s ability to respond to and influence male courtship, and therefore the male largely relies upon pheromonal information to judge a female. In light conditions, only courtship index was affected (Two-way ANOVA, courtship latency: *F*_3,115_* = *3.0558, *P = *0.0313, Fig. 1C; courtship index: *F*_3,124_* = *6.3870, *P = *0.0005, Fig. 1G, Supplemental Table 2; Bonferroni corrected α = 0.025), yet post-hoc Tukey’s tests did not reveal differences in either control or HFD male behavior. However, control males significantly changed their courtship behavior towards decapitated 3% HFD females in the dark condition while HFD males did not (Two-way ANOVA, courtship latency: *F*_3,98_* = *3.8501, *P = *0.0120, Fig. 1D; courtship index: *F*_3,109_* = *3.9099, *P = *0.0108, Fig. 1H, Supplemental Table 2; Bonferroni corrected α = 0.025). Together, these assays provide support for the hypothesis that HFD altered the female pheromone profiles that control males use to assess female attractiveness.

### Adult-only High Fat Diet Affects Mating Behavior in a Similar Manner as Developmental High Fat Diet

To further remove the confounding effect of HFD on female body size on male mate assessment, we removed the developmental influence of HFD and examined adult-only dietary effects by collecting newly eclosed flies that had developed on a common diet (control diet) and then transferring these adult flies to either the control diet or to diets containing a range of increased fat (3%, 7%, 15% or 30% adult-only diet), expecting that an increased fat dosage during adulthood would be necessary to phenocopy the effects of the 3% developmental diet. Similar effects on female attractiveness from the developmental and adult-only diet treatments will indicate that HFD likely affects sexual cues such as pheromone profiles. Because few females survived the 30% diet, we only evaluated how this diet affected male behavior and attractiveness.

Mating behaviors for intact male and female pairs were examined in light conditions only. Each adult-only diet treatment affected *D. melanogaster* mating behavior (One-way MANOVA: 3% HFD, Wilks’ Lambda = 2.2117, *P = *0.0119; 7% HFD, Wilks’ Lambda = 5.2753, *P < *0.0001; 15% HFD, Wilks’ Lambda = 5.4934, *P < *0.0001; 30% HFD, Wilks’ Lambda = 6.8127, *P = *0.0002). The 3% and 7% adult-only diets had similar effects on female *Drosophila* (decreased mating success and activity levels) but did not recapitulate the loss of female attractiveness that was observed with developmental 3% HFD (Supplemental Fig. 2 and Supplemental Tables 3 and 4).

**Figure 2:**
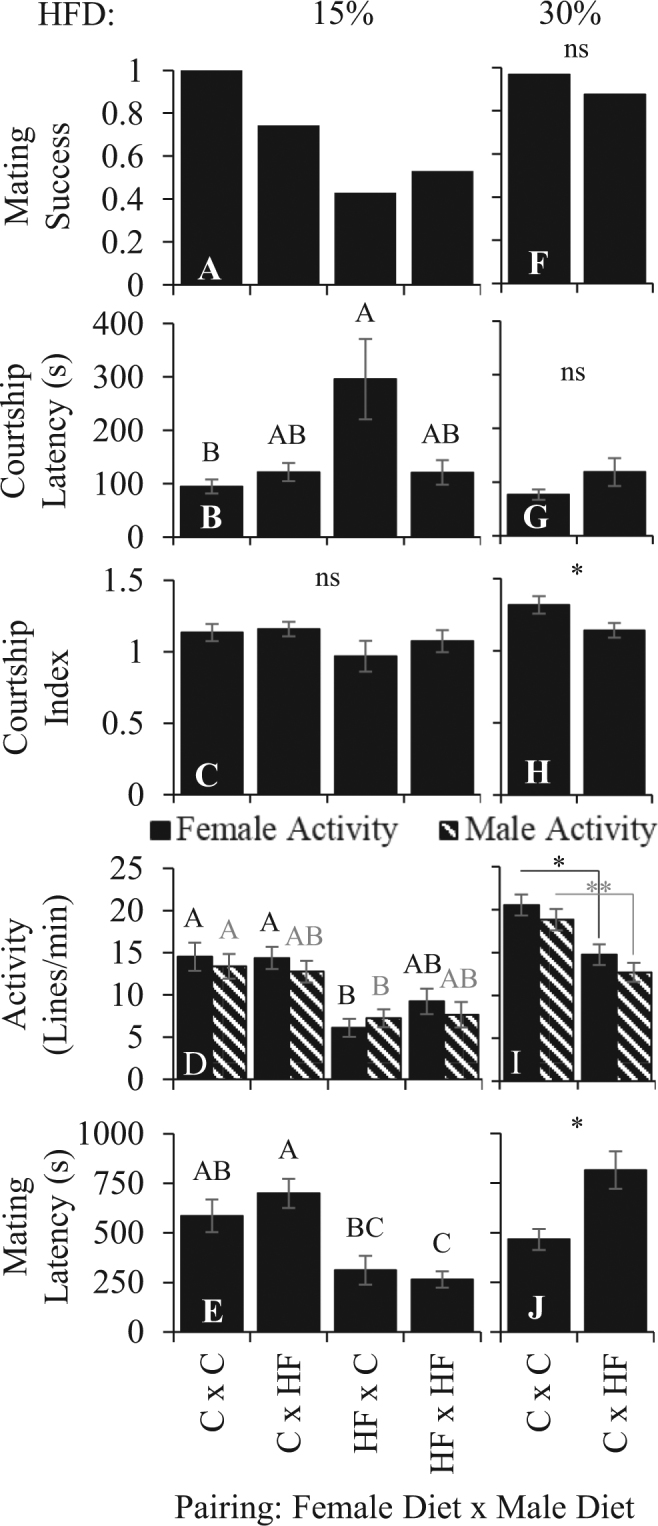
Effects of adult-only HFD on behavior. The effect of 15% (**A**–**E**) or 30% (**F**–**J**) adult-only HFD on *D. melanogaster* mating behavior. Pairings are female diet X male diet. Each bar represents the mean ± SE of N = 25. The letters above the bars (**A**–**E**) represent a post-hoc Tukey’s HSD where means that do not share the same letter are significantly different at Bonferroni corrected α = 0.01. Means with * and ** (**F**–**J**) were significantly different (*P* = 0.01 and 0.001, respectively). ns = not significant.

In contrast, the mating behaviors of flies provided 15% HFD only during adulthood approximated most phenotypes that were observed from the developmental 3% HFD (Fig. 2 and Supplemental Table 5). Mating success decreased (Chi square test, χ^2^ (3, *N* = 104) = 28.437, *P* < 0.0001, Fig. 2A), and 15% adult-only HFD affected courtship latency (Two-way ANOVA, *F*_3,103_ = 4.1775, *P* = 0.0079; Fig. 2B) but not courtship index (Fig. 2C). For courtship latency, only the interaction term between female and male diet was significant (*F* = 7.1324, *P = *0.0088), and the Tukey’s post-hoc test revealed that control males took longer to begin courting HFD females than control females. Similar to all other HFD treatments, activity levels (Two-way ANOVA: Female activity, *F*_3,101_ = 8.89851, *P < *0.0001; Male activity, *F*_3,101_ = 5.2609, *P = *0.0021; Fig. 2D) and mating latency (Two-way ANOVA, *F*_3,98_ = 10.8396, *P < *0.0001; Fig. 2E) were affected by 15% HFD, where only the female diet term was significant (Female activity: *F* = 24.0652, *P < *0.0001; Male activity: *F* = 15.2366, *P* = 0.0002; Mating latency: *F* = 31.2037, *P < *0.0001). Overall, 15% HFD females were less active, mated more quickly, and were courted less quickly by control males.

The 30% HFD did not affect male mating success (Fig. 2F and Supplemental Table 6) or courtship latency (Fig. 2G), but courtship index (*t*-test, *t*_43.5566_ = −2.7007, *P* = 0.0096, Bonferroni corrected α = 0.01; Fig. 2H), activity levels (Female activity: *t*-test, *t*_51.7931_ = −3.4085, *P* = 0.0013; Male activity: *t*-test, *t*_52.0143_ = −3.7063, *P* = 0.0005; Figure I), and mating latency (*t*-test, *t*_52.3464_ = 2.9586, *P* = 0.0046; Fig. 2J) were altered. 30% HFD males courted females less and were less active than control males. Control females paired with these HFD males were less active than when paired with control males and took longer to mate with 30% HFD males. These results suggest that 30% HFD males are less attractive.

To confirm that 30% HFD decreases male attractiveness, a phenotype not detected with 3% developmental HFD or lower dose adult-only diets, the competitive ability of both 15% HFD and 30% HFD males was examined (Table 1). When 15% HFD males competed with control males for a control female, the 15% HFD males performed courtship behaviors similarly to control males and achieved a similar number of matings. The results of this competition assay match the results of the single-pair mating assays where the mating behavior of control females did not indicate aversion to 15% HFD males. Yet when 30% HFD males competed against control males for control females, the HFD males achieved fewer matings (Chi-square test, χ^2^ (1, *N* = 50) = 11.796, *P* = 0.0006) despite courting similarly. These competition results provide further evidence that control females find 30% HFD males less attractive.

**Table 1:** Male competition for control and high fat (15% or 30%) adult-only diets.

Female	Males	Behavior	Result	Statistic	*P*
Control	Control vs 15% High Fat	Courtship Latency	C = 1.88 ± 0.04 HF = 1.93 ± 0.05	t = 0.7497	0.4558
		Courtship Index	C = 0.76 ± 0.04 HF = 0.70 ± 0.04	t = 0.9423	0.3491
		Mated	C = 60% HF = 40%	χ^2^ = 3.20	0.0736
Control	Control vs 30% High Fat	Courtship Latency	C = 1.79 ± 0.05 HF = 1.70 ± 0.05	t = 1.4483	0.1508
		Courtship Index	C = 0.82 ± 0.03 HF = 0.89 ± 0.03	t = 1.6623	0.0999
		Mated	C = 67.3% HF = 32.7%	χ^2^ = 11.796	**0.0006**

Values in bold are significant at Bonferroni corrected α = 0.0167.

### High Fat Diet Affects CHC Profiles

To determine if pheromone profiles are impacted by HFD, we quantified individual CHCs of females provided either control, 3% developmental, or 3% or 15% adult-only HFD. We also quantified male CHCs from flies raised on these same diets as well as the 30% adult-only HFD.

**Figure 3:**
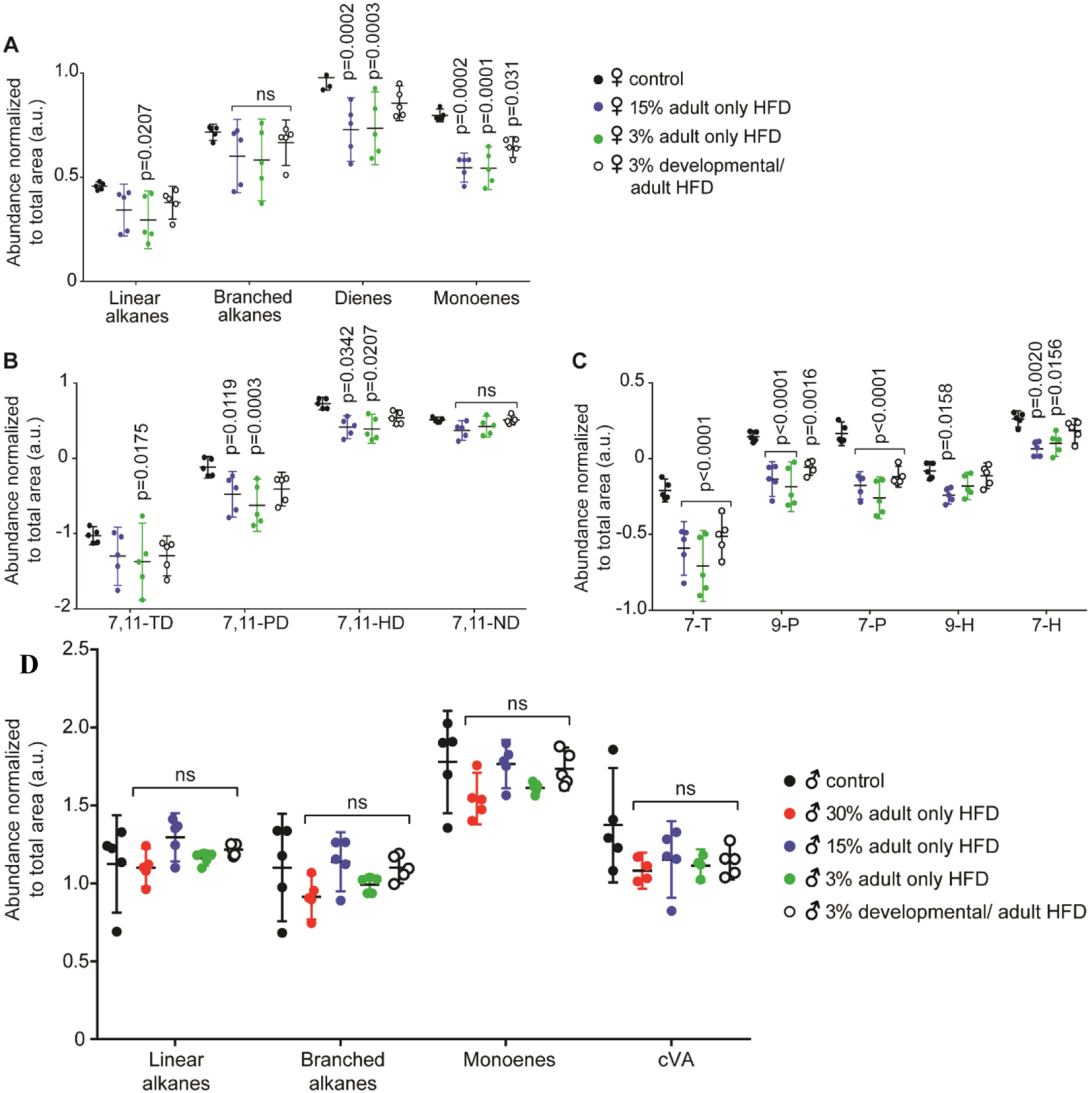
HFD affects female but not male CHC profiles. The effect of developmental and adult-only HFD on CHC chemical categories (**A**), or individual dienes (**B**) or monoenes (**C**) in females and males (**D**). Each experimental condition was compared to the control diet; p-values obtained with a post-hoc Bonferroni’s multiple comparisons test are shown; unlabeled treatments are not significantly different. The mean (middle bar) with 95% confidence interval is shown and each point represents each replicate (N = 5 with 8 flies per replicate); ns: not significant, TD: tricosadiene, PD: pentacosadiene, HD: heptacosadiene, ND: nonacosadiene, T: tricosene, P: pentacosene, H: heptacosene, cVA: cis*-*vaccenyl acetate.

In females, the CHCs were more affected in the 3% and 15% adult-only HFD than in the 3% developmental HFD (Fig. 3 and Supplemental Fig. 3). HFD altered different categories of CHCs (Fig. 3A), with dienes and monoenes being the most strongly affected by adult-only HFD conditions. Surprisingly, the developmental HFD only altered monoene levels. Amongst the dienes (Fig. 3B), both 7,11-pentacosadiene and the female aphrodisiac 7,11-heptacosadiene decreased in adult-only HFD conditions while 7,11-tricosadiene only decreased in adult-only 3% HFD females. Monoenes including 7-tricosene, 7-and 9-pentacosene and 7-heptacosene were similarly affected by adult-only HFD regime. The 3% developmental diet led to a reduction of 7-tricosene and 9-pentacosene (Fig. 3C).

Male CHC profiles were not altered by HFD (Fig. 3D and Supplemental Fig. 4).

### High Fat Diet Does Not Significantly Affect Male Courtship Song

Next, we compared courtship singing between control diet and 30% adult-only HFD males. We found no significant difference in the percentage of pulse song produced during courtship. HFD males devoted 5.40% of their time to pulse song compared to the control group’s 8.76%. While high variance among the individual males precludes our ability to identify a significant effect of diet on the percentage of time males spent performing pulse song, we did observe a general trend of the control diet males pulse singing longer and for a greater proportion of the total recorded time.

### Metabolic Rescue

As physiological defects caused by HFD are mediated by highly conserved metabolic signaling pathways^35,40^, we hypothesized that genetic manipulations that have been shown to rescue negative health impacts of HFD may also rescue behavioral defects. To test this prediction, we examined the effects of three different genetic manipulations of *Drosophila* metabolic pathways: overexpression of FOXO (to suppress insulin signaling) and Bmm (to increase fat lipase signaling) and expression of TOR^DN^ (to decrease TOR signaling).

**Figure 4:**
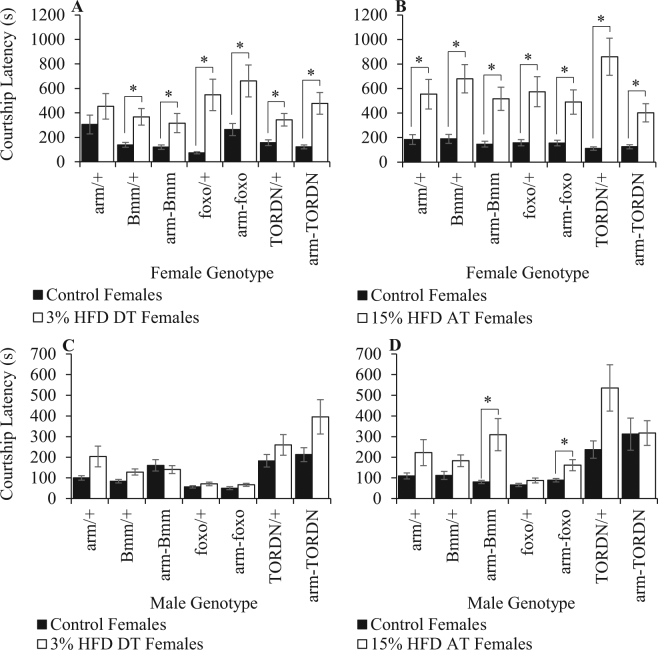
Genetic manipulation of metabolic signaling pathways. The ability of ubiquitous expression of *UAS-Bmm, UAS-foxo*, or *UAS-TOR*^*DN*^ to rescue developmental or adult-only HFD effects on female attractiveness (**A**,**B**) and male mate assessment (**C**,**D**). In panels A and B, all males are *CS* males raised on control diet. The male diet was manipulated in panels C and D: 3% developmental diet treatment (DT) males (**C**) and 15% adult-only diet treatment (AT) males (**D**). Each bar represents the mean ± SE of N = 30. Means with * were significantly different with a Bonferroni corrected α = 0.025.

We first tested the ability of metabolic manipulations in females to rescue female attractiveness. We expected wild-type males raised on control diet to have increased courtship latencies toward genetic control (*arm-Gal4/* + , *UAS-Bmm/* + , *UAS-foxo/* + , or *UAS-TOR*^*DN*^*/* + ) females provided HFD compared to similar females provided control diet. However, if female overexpression of FOXO, Bmm, or TOR^DN^ rescues female attractiveness, male courtship latencies should not differ significantly toward control diet and HFD females of the rescue genotypes (*arm-Gal4/UAS-Bmm, arm-Gal4/UAS-foxo*, and *arm-Gal4/TOR*^*DN*^). We found that control diet males have significantly increased mating latencies toward females (both the control and rescue genotypes) that were given developmental (Fig. 4A) or adult-only HFD (Fig. 4B) treatments, indicating that these genetic manipulations failed to rescue female attractiveness.

We next asked whether similar genetic manipulations of metabolic pathways in HFD males could restore the ability of these males to discriminate between control and HFD females. If so, we expected that HFD males with rescue genotypes (*arm-Gal4/UAS-Bmm, arm-Gal4/UAS-foxo*, and *arm-Gal4/TOR*^*DN*^) would have increased courtship latencies toward wild-type HFD females compared to control diet females. All genotypes of males provided 3% developmental diet had similar courtship latencies toward control and HFD females (Fig. 4C). However, 15% adult-only HFD males that overexpress Brummer lipase (*arm-Gal4/UAS-Bmm*; Two-tailed student’s *t*-test: *t* = −5.32692, *P* < 0.0001) or FOXO (*arm-Gal4/UAS-foxo*; Two-tailed student’s *t*-test: *t* = −2.68428, *P* = 0.0097) took longer to begin courting HFD females, while their genetic controls (*arm-Gal4/* + , *UAS-Bmm/* + , and *UAS-foxo/* + ) had similar courtship latencies toward control diet and HFD females (Fig. 4D). Overexpression of Bmm and FOXO are therefore able to rescue adult-specific HFD effects on male assessment of female attractiveness.

## Discussion

Similar to effects in other animals, overconsumption of lipids has severe detrimental effects on the health of *D. melanogaster*^35,40^, and this decrease in fly health is correlated with alterations in mating behavior^42^. In females, developmental HFD leads to decreased activity levels, body size, and fecundity, all of which reflect the worsened condition of the fly and ultimately lead to decreased female attractiveness. Therefore, some female sexual trait(s) evaluated by males during courtship must also be altered by HFD. Male flies are more resilient to the effects of HFD, as developmental HFD does not decrease male body size, activity levels, or attractiveness, but exposure to developmental HFD does affect the male’s ability to discriminate between poor and good condition mates^42^. Here, we have expanded our understanding of how HFD affects *D. melanogaster* mating behavior by identifying dietary conditions that decrease male and female attractiveness by interrogating which sexual traits have been modified by HFD, and whether changes in mating behavior and attractiveness are mediated by conserved metabolic signaling pathways.

### Developmental Exposure to High Fat Diet Alters Nonvisual Female Sexual Traits

The major sexual traits that signal female attractiveness are body size, behavioral responses to male courtship, and pheromone profiles^44^. *Drosophila* males prefer larger females^25^, and female acceptance or rejection behaviors also provide feedback to courting males, leading them to increase or decrease their courtship efforts^44^. We previously demonstrated that HFD decreased female activity levels prior to mating^42^, which may be an indication of increased receptivity^45^. However, it is possible that less mobile females are judged by males as being less attractive. Pheromones are another important sexual cue that convey information about species, sex, age, and mating status^46,47^. In particular, the long-chain hydrocarbon dienes 7,11-heptacosadiene and 7,11-nonacosadiene, characteristic of female cuticles, function as aphrodisiacs for males^48–50^. Dietary sugar and protein levels are known to alter *D. melanogaster* pheromone profiles^51^, so it is likely that dietary lipids also affect pheromone production, especially since pheromone biosynthesis partially relies upon precursors from diet-derived fatty acids^52^. Therefore, there are a number of sexual traits that could be modified by HFD to decrease female attractiveness, and we hypothesized that pheromone changes are important contributors.

To demonstrate that HFD alters female pheromone profiles in a way that decreases female attractiveness, we examined males as they courted HFD females without the confounding effects of diet on female body size and behavior. Control males discriminated against HFD females in both light and dark conditions by delaying the start of courtship, indicating that nonvisual sexual cues are important for male judgment of female quality. Although we observed expected changes in courtship latency in the light and the dark and in courtship index in the light, where control males decreased performed less courtship towards HFD females, control male courtship index towards HFD females was not altered in the dark. In fact, courtship indices for all pairings in the dark with intact females were drastically reduced. During video analysis, it was clear that males could not accurately track females in the dark, resulting in sporadic courtship bursts as flies came in contact in the courtship chambers. The general difficulty in finding mates in the dark may explain why there was no decrease in control male courtship towards intact 3% HFD females.

The light or dark condition assays were repeated with decapitated females, which will not respond to courtship advances, allowing for the removal of female behavioral feedback from male mate assessment. Additionally, decapitated females are less mobile, which should allow males to find females more easily in dark conditions. We found that control males did not modify their behavior significantly towards decapitated HFD females in the light. In light conditions, males can visually examine the decapitated females, so that although they stand upright, the females may have appeared injured or the males may have noted their inactivity, leading to increased courtship latency and decreased courtship index towards all types of decapitated females compared to intact females. Such judgments cannot be made in dark conditions, where we observed that control males increased courtship latency and decreased courtship index toward HFD females. These experiments demonstrate that control males discriminated against HFD females without visual assessment of body size or behavioral feedback from the female, indicating that changes in pheromonal profiles likely contribute to male discrimination against 3% HFD females.

### Adult-Only Exposure to Increased High Fat Diet Dosage Recapitulates Female Phenotypes Caused by Developmental Exposure

Developmental exposure to environmental factors can have dramatic, long-lasting effects on fly health. Larval crowding or malnutrition can cause numerous alterations in fly traits, including body size^24,53–56^. To fully remove the potential confounding effect of HFD on fly body size, we raised the flies on a similar control diet during development and only exposed the adults to increasing amounts of fat. We found that all levels of HFD caused changes in female activity levels, but the behavioral defects seen in the developmental treatment were only recapitulated with the 15% adult-only diet treatment. At this level, HFD females mated faster, indicating that they are less choosy or more receptive to mating. Control males took longer to begin courting HFD females, indicating that the 15% HFD females are less attractive due to changes in a trait other than body size.

Interestingly, control males did not decrease their overall courtship effort towards the adult-only HFD females as they had towards the developmental HFD females. It is therefore possible that common changes in pheromones caused by the developmental and adult-only diets are important for initial male assessment of the female and influence his decision to begin courtship behavior, i.e. to enter an arousal state^57^. Once in this state, visual assessment of smaller developmental HFD females may have led males to decrease courtship, which would not happen with the adult diet females where body size is unaltered. Yet, males decreased courtship toward decapitated developmental HFD females in the dark when visual assessment was not possible.

### High Fat Diet Alters Female Pheromones

Both the developmental and adult-only HFD caused similar decreases in the levels of cuticular monoenes and dienes (primarily 7,11-heptacosadiene) of females. Additionally, the overall amount of CHCs produced by adult-only HFD treated females, but not developmental HFD females, decreased. These differences may be due to larval adaptation to HFD, leading to changes in adult metabolism and responses to HFD during adulthood, as has been observed in *D. melanogaster* in response to other types of larval dietary stress^58^. The loss of the aphrodisiac 7,11-heptacosadiene in females under the 3% developmental and 15% adult-only HFD regimes is consistent with a loss of attractiveness. However, while 7,11-heptacosadiene levels also decreased in the 3% adult-only HFD females, a corresponding change in female attractiveness was not observed, suggesting that although a change in pheromone levels may contribute to diet-induced alterations in attractiveness, a combination of sensory features may be used by males in the decision to court. Future experiments using CHC extracts from 15% HFD females to “perfume” oenocyte-less flies may provide an avenue for direct tests of whether diet-induced changes in CHCs underlie the loss of female attractiveness.

### Adult-Only Exposure to Increased High Fat Diet Dosage Recapitulates Male Phenotypes Caused by Developmental Exposure

Neural circuits necessary for *D. melanogaster* mating behavior are established during development^59–61^. For example, expression of *ecdysone receptor* (*EcR*) in *fruitless* (*fru*) expressing neurons is necessary for standard male courtship behavior. Reduction of *EcR* expression in *fru* neurons during adulthood has little effect on male courtship behavior, but disruption during development increased male courtship performance^62^. However, reduced *EcR* signaling during adulthood in has been shown to increase male-male courtship^63^. We previously observed that males exposed to HFD during development did not find HFD females unattractive, as control males had, and did not appear to distinguish between control and HFD females to any degree^42^. HFD therefore appears to alter male physiological functions that are important for mate discrimination, resulting in less choosy males. Whether exposure to HFD during development is necessary for this alteration to occur, or whether the change could happen after neural circuits underlying courtship behavior are established is unknown. We addressed this question by raising flies on a common, control diet and then exposing the flies to increasing amounts of HFD in adulthood. Courtship latency of 15% HFD males towards 15% HFD and control females did not differ, even though control males found 15% HFD females unattractive. This result suggests that HFD does not necessarily affect neural development to alter male discrimination ability, but that HFD may affect neural function post-development instead. This possibility could be evaluated by examining the activity of pheromone-responsive neurons^57,64^ in HFD males compared to control males. In control males, these neurons would be expected to have lower activity when stimulated by unattractive pheromone profiles from HFD females, whereas in HFD males similar activity would be expected in response to both control and HFD profiles.

### Only an Extreme Dosage of High Fat Diet Decreases Male Attractiveness

Males appear to be more resistant to dietary lipid effects than females, as only changes in male mate perception occurred at lower fat doses, while a multitude of behavioral defects were seen in females treated with the same dose. Sex-specific physiology and responses to environmental factors are well documented in *D. melanogaster*. For example, females live^65^ and withstand starvation longer^66^, and exercise only benefits male flies^67^. While the 3% developmental and 15% adult-only HFD treatments decreased female attractiveness, these same treatments had no effect on male attractiveness. Male attractiveness was affected only when males were fed 30% adult-only diet. Both competitive and non-competitive assays revealed that 30% males were less attractive to control females. However, no differences in pheromone levels were identified in HFD males, and while our song analyses did not identify a significant effect of male HFD on courtship song performance, there was a trend toward HFD males performing less pulse song than control diet males. Therefore, it is possible that a song that is slightly poorer in quality due to diet has some influence on how the female perceives the male.

### Metabolic Signaling Rescue of High Fat Diet Effects on Mating Behavior

Our understanding of how HFD affects *D. melanogaster* mating behavior centers on the idea that HFD alters fly physiology and health, which affects sexual traits, leading to changes in mating behavior. This argument would be strengthened if blocking the HFD effects on fly health rescues the effects of HFD on mating behavior and attractiveness. Upon exposure to HFD in wild-type flies, insulin and TOR signaling initially increase. Genetic manipulations that either block this initial induction of the insulin/TOR pathway or cause overexpression of a fat lipase, Brummer (Bmm), rescue health defects caused by HFD^35,40^. Insulin signaling results in the repression of *foxo* expression and de-represses TOR signaling by deactivating TOR inhibitors. Overexpression of FOXO and a dominant-negative version of TOR (TOR^DN^) therefore serve to decrease the downstream components of insulin signaling. Increased Bmm expression leads to increased breakdown of stored lipids, negating the effects caused by lipid accumulation. Genetic manipulations of conserved metabolic signaling pathways (insulin, TOR, and Brummer) have been shown to rescue certain HFD physiological defects, such as heart dysfunction, lipid accumulation, and insulin resistance^35,40^, and female pheromone profiles and attractiveness are also regulated by insulin and TOR signaling^32^. We performed the same genetic manipulations on both developmental and adult-only HFD treatments, using the same Gal4 driver, to determine whether HFD effects on behavior and attractiveness could be rescued. No developmental HFD treatment defects were rescued, which may not be surprising as only adult-specific defects were examined and rescued by Birse *et al*. (2010). Yet when evaluating adult-specific dietary effects on behavior, we found that while female attractiveness was not rescued by any genetic manipulation, FOXO and Brummer overexpression rescued male discrimination ability. TOR-DN expression did not alter male phenotypes, possibly because TOR signaling induces a separate genetic cascade than FOXO signaling that may not be involved in male mate discrimination. These results provide evidence that repression of insulin signaling effects by overexpression of FOXO, which is inhibited by insulin signaling, and blocking fat accumulation by overexpression of Brummer, a fat lipase, can rescue male health sufficiently to rescue male-specific HFD behavioral defects but not female-specific effects. However, these results do not eliminate the possibility that conserved metabolic signaling pathways are involved in mediating the female response to HFD. HFD affects females more strongly than males, and the female-specific effects may be too severe to rescue with these genetic manipulations. Even though certain physiological defects caused by HFD can be rescued, female activity levels were not^35^, and whether these manipulations rescue other defects, such as mortality, has not yet been examined. It is possible that the driver used in these studies (*armadillo-Gal4*) does not provide high enough expression to rescue drastic physiological defects caused by HFD that lead to the observed alterations in females.

## Conclusion

In conclusion, our study shows that HFD affects *D. melanogaster* behavioral interactions through alteration of both visual and non-visual sexual traits, and that females are more susceptible to defects caused by HFD. The less severe effects of HFD on males can be rescued by genetic manipulation of conserved metabolic signaling pathways. Further characterization of HFD impacts on *D. melanogaster* behavior could advance the understanding of how genetic and environmental factors interact to affect animal health and sexual selection^43^, drawing together two often disparate fields of research^3^ to provide insights into the fluidity of sexual selection.

## Methods

### Fly Stocks

All *Canton-S* (*CS*) wild-type flies used in this study were isogenized by backcrossing sibling pairs for 10 generations. *arm-Gal4* (w[*]; P[w[ + mW.hs] = GAL4-arm.S]11), *UAS-TOR*^*DN*^ (y^1^ w*; P[UAS-Tor.TED]II), and *UAS*-*FOXO* (w[1118]; P[[w + mC] = UASp-foxo.S]3)^35^ were obtained from the Bloomington *Drosophila* Stock Center. *UAS-Bmm* was a gift from Dr. Rolf Bodmer at the Sanford/Burnham Medical Research Institute. Each stock was outcrossed 6 × into the *CS* isogenic background. All stocks were maintained on standard lab food (10 g/L *Drosophila* agar, 40 g/L dextrose, 20 g/L sucrose, 12 g/L nutritional yeast, 70 g/L cornmeal, 3 ml/L of 10% Tegosept), and flies used in the behavioral experiments were raised in bottles containing either control diet (C: 7 g/L agar, 65 g/L cornmeal, 13 g/L inactive yeast, 7.5 g/L sucrose) or high fat diet (3% HFD: C + 30 g/L coconut oil; 7% HFD: C + 70 g/L coconut oil; 15% HFD: C + 150 g/L coconut oil; 30% HFD: C + 300 g/L coconut oil)^26,35,42,68^ and maintained in food vials (5 females/vial; 1 male/vial) as virgins until testing^42^.

### Body length measurements

The movements of pairs of *CS* male and female flies raised on C or 3% HFD (*N = *100 for each sex on each diet) were video recorded in 1 cm diameter plexiglass courtship chambers. Three separate still frames were captured from the videos during periods when the flies were walking with straight abdomens in a non-angled orientation. Body length was measured from the tip of the head to the tip of the abdomen in ImageJ, and the three measurements per fly were averaged to give a body length measurement.

### Behavioral testing: General protocol

For single-pair mating assays, one virgin female and male were placed in a 1 cm diameter courtship chamber with a moistened filter paper. Pairings consisted of two control diet flies, one C female and one HFD male, one HFD female and one C male, or two HFD flies (i.e., four types of pairings). For male competition assays, one C female and two males (one C and one HFD) were placed in the courtship chambers. Interactions were recorded for 1 hr with high definition video cameras.

Videos were later analyzed by an observer who was blind to the fly diets. Mating success (the proportion of successful matings out of the total number of pairs evaluated^69–71^), courtship latency (the amount of time from introduction until males begin courting; indicates male assessment of female attractiveness^71–75^), courtship index (the proportion of time the male spent courting until the beginning of mating; also indicates assessment of female attractiveness^71,76^), activity levels in the minute prior to mating (indicative of male condition and female receptivity^45,73,77^), and mating latency (the amount of time from the beginning of courtship until mating begins; indicates female assessment of male attractiveness^71,74,75,78,79^) were quantified as described previously^42^.

### Behavioral testing: Developmental diet treatment and light vs. dark conditions

*CS* flies were raised and maintained post-eclosion on C diet or 3% HFD and paired in a fully combinatorial manner as described above. Male courtship and mating behaviors were observed in both light and dark conditions (chambers were illuminated with red light in order to record behaviors) with intact, freely behaving females or with decapitated females. Female decapitation occurred immediately before the courtship assays, which were initiated once females recovered from CO_2_ anesthesia. The light vs. dark experiments were only performed on the developmental 3% diet females because, with this treatment, the only way to decouple the effects of diet on body size from other sexual traits is to eliminate the male’s ability to see the female. In order to determine how male perception of females varied with exposure to HFD and the absence or presence of light, mating latency and courtship index were measured.

### Behavioral testing: Adult-only diet treatment

Wild-type *CS* flies were raised on C diet and transferred upon eclosion to vials containing either C food or diets with a range of increased fat content (3%, 7%, 15%, or 30%). For single-pair mating assays, separate experimental blocks for each percentage of HFD were performed in a fully combinatorial manner, except for experiments with the 30% HFD because high female mortality necessitated testing males only (two pairings: C female with C male, C female with 30% HFD male). In these assays, mating success, courtship latency, courtship index, activity levels, and mating latency were quantified. Competition assays were performed in which a C diet male and a HFD male (15% or 30% HFD) competed for matings with a C female in order to confirm the effect of 30% HFD on male attractiveness.

### Cuticular hydrocarbon analysis

Male and female flies were provided C or HFD (3% developmental diet, 3% adult-only diet, 15% adult-only diet, or 30% adult-only (males only)) and handled as described above. The 7% adult-only diet was excluded as no behavioral phenotypes were observed with this diet. The 3% adult-only diet, which also had little effect on behavior, was included in order to compare developmental and adult-only treatment with the same HFD dosage. Cuticular hydrocarbons (CHCs) were extracted from five-day-old animals as described previously^51^. Briefly, five replicates of eight flies from each treatment group were incubated in 120 μL hexane spiked with 10 μg/mL hexacosane (Sigma-Aldrich; St. Louis, Missouri, USA) for 10 min at room temperature after brief vortexing. 100 μL of hexane were removed and put in a new vial, and the hexane was allowed to evaporate for 4–6 hours. Vials were stored at −20 °C until analysis.

Gas chromatography mass spectrometry (GCMS) analysis was performed on a 7820 A GC system equipped with a 5975 Mass Selective Detector (Agilent Technologies, Inc., Santa Clara, CA, USA) and a HP-5ms column ((5%-Phenyl)-methylpolysiloxane, 30 m length, 250 μm ID, 0.25 μm film thickness; Agilent Technologies, Inc.). Electron ionization energy was set at 70 eV. One microliter of the sample was injected in splitless mode and analyzed with helium flow at 1 mL/ min. The following parameters were used: column was set at 40 °C for 3 min, increased to 200 °C at a rate of 35 °C/min, then increased to 280 °C at a rate of 20 °C/min for 15 min. The MS was set to detect from *m/z* 33 to 500. Chromatograms and spectra were analyzed using MSD ChemStation (Agilent Technologies, Inc.). The abundance of each CHC species was calculated by i) normalizing the area under each CHC peak to the total area of all CHC peaks for relative ratios and ii) integrating the area under the CHC peak and normalizing to the hexacosane internal standard. Values obtained for relative ratio calculations were transformed using a log contrast function, with the nC27 peak used as the divisor^80,81^.

### Song recording and analysis

One 5-day-old C diet or 30% adult-only HFD male was placed with a C diet female into a courtship song recording chamber, which was a modified 1.5 mL microcentrifuge tube cut to a 5 cm height and sealed with mesh. The mesh side of the courtship song recording chamber was placed directly on top of the microphone in an insect recording box that was built based upon the Insectavox design^82^. An LED light within the box provided light during courtship. All songs were recorded in a quiet room with foam lined walls at room temperature (~25 °C). Courtship song was recorded using Raven Lite: Interactive Sound Analysis Software from the Cornell Lab of Ornithology Bioacoustics Research Program. Males were allowed to court females for 5 min or until mating occurred. *N = *10 for each male diet.

We evaluated songs from each male for the percentage of time the male spent on pulse song during the evaluation period (5 min or until the final pulse before mating). We considered only continuous pulse trains with 3 or more pulses. Trains were deemed discontinuous if the space between them is > 2*a* (where *a* = the interpulse interval of the former train)^30^. To calculate the percentage of time each male spent singing, the pulse song duration was divided by the total evaluation period and averaged across all males for that diet.

### Behavioral testing: Metabolic rescue

*UAS-TOR*^*DN*^, *UAS-Bmm*, and *UAS-FOXO* were expressed ubiquitously with *arm-Gal4*^35^. Flies containing *arm-Gal4* together with a UAS expression construct (rescue genotypes) were compared to their genetic controls (animals containing single components: *arm-Gal4, UAS-TOR*^*DN*^, *UAS-Bmm*, or *UAS-FOXO*) for effects on their ability to rescue phenotypes resulting from HFD (decreased female attractiveness; male discrimination ability). To test for rescue of developmental diet effects, flies containing the UAS or Gal4 transgenes or both constructs were raised on 3% HFD or C food as before. To test for rescue of adult-only HFD effects, flies were raised on C diet and transferred to C or 15% HFD upon eclosion as described previously.

C diet or HFD (3% developmental diet or 15% adult-only diet) females of the rescue or genetic control genotypes were placed in single-pair mating assays with *CS* males raised on C diet, and pairs were video recorded. Male courtship latency was measured to evaluate female attractiveness.

To test for rescue of male discriminatory ability, HFD males (3% developmental diet or 15% adult-only diet) of rescue or genetic control genotypes were placed in single-pair mating assays with C or HFD (3% developmental diet or 15% adult-only treatment) *CS* females and video recorded. Male courtship latency towards these females was then quantified.

### Statistics

Normal distribution of logarithmic (courtship latency and mating latency) and arcsine transformed (courtship index) data was confirmed with the Shapiro-Wilk test.

To determine the necessity of male visual assessment or female behavior for the observed effects of HFD developmental treatment on male reproductive behavior, the courtship latency and courtship index for each experiment (light condition assays with intact females, dark condition assays with intact females, light condition assays with decapitated females, or dark condition assays with decapitated females) were examined with a two-way ANOVA (y = female diet + male diet + female*male diet). A Bonferroni correction of α = 0.025 was applied to control for testing dietary effects on two behavioral traits.

To examine the effects of adult-only HFD treatment, statistical analysis followed the procedures described previously^42^. A one-way MANOVA was performed with all behavioral parameters for each HFD dosage (3%, 7%, and 15%) to first determine whether diet had any effects on the traits. Each dietary level was then tested with a two-way ANOVA for each behavioral trait (y = female diet + male diet + female*male diet) for post-hoc analysis of significance. Finally, a Bonferroni correction of α = 0.01 was applied to control for multiple testing. Mating success was analyzed via chi-square tests.

Competition assay data were analyzed as described previously^42^. The proportion of control or HFD males that gained matings was analyzed with a chi-square test, while courtship latency and courtship index were analyzed with a *t-*test. A Bonferroni correction of α = 0.0167 was applied to control for testing dietary effects on three behavioral traits.

For metabolic rescue experiments, *t-*tests were performed on the behavioral data from the control diet and the HFD treatment of each genotype for the examination of female attractiveness, and on the behavioral data of the HFD male towards control and HFD wild-type females for the examination of male mate assessment. A Bonferroni correction of α = 0.025 was applied to control for testing developmental versus adult-only effects.

For CHC analysis, a two-way ANOVA was used to compare abundances of individual CHC chemical categories and multiple comparisons corrected for using the Bonferroni correction (GraphPad Prism 5, Graph Pad Software Inc., CA, USA).

### Data availability statement

The datasets generated during and/or analyzed during the current study are available from the corresponding author on reasonable request.

## Electronic supplementary material

Supplemental Information
