# Supplementary material for: High fat diet alters Drosophila melanogaster sexual behavior and traits: decreased attractiveness and changes in pheromone profiles
Source: Sci Rep. 2018 Mar 29;8:5387. doi: 10.1038/s41598-018-23662-2 (PMC5876352; doi:10.1038/s41598-018-23662-2)
Supplement: Supplementary file 1 — Supplemental Information [file 41598_2018_23662_MOESM1_ESM.pdf]

Supplementary information

High fat diet alters *Drosophila melanogaster* sexual behavior and traits: decreased attractiveness and changes in pheromone profiles

Janna N. Schultzhaus, Chloe J. Bennett, Hina Iftikhar, Joanne Y. Yew, Jason Mallett, & Ginger E. Carney

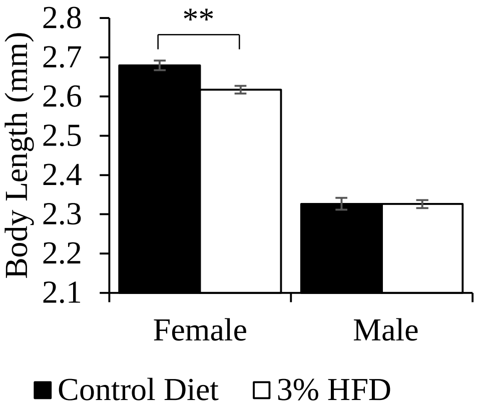

Supplemental Figure 1. Developmental HFD decreases female, but not male, body size.

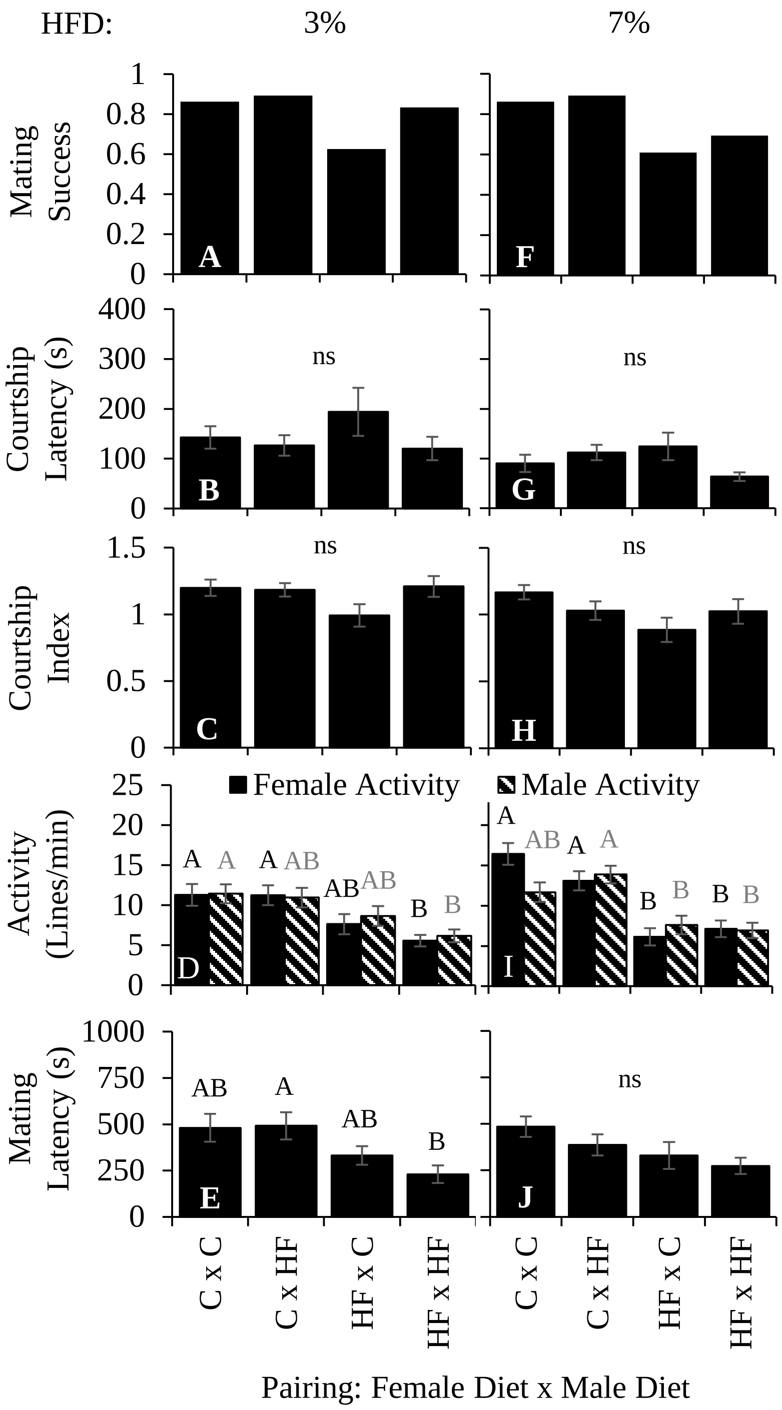

Supplemental Figure 2. Effects of adult-only 3% or 7% HFD on behavior.

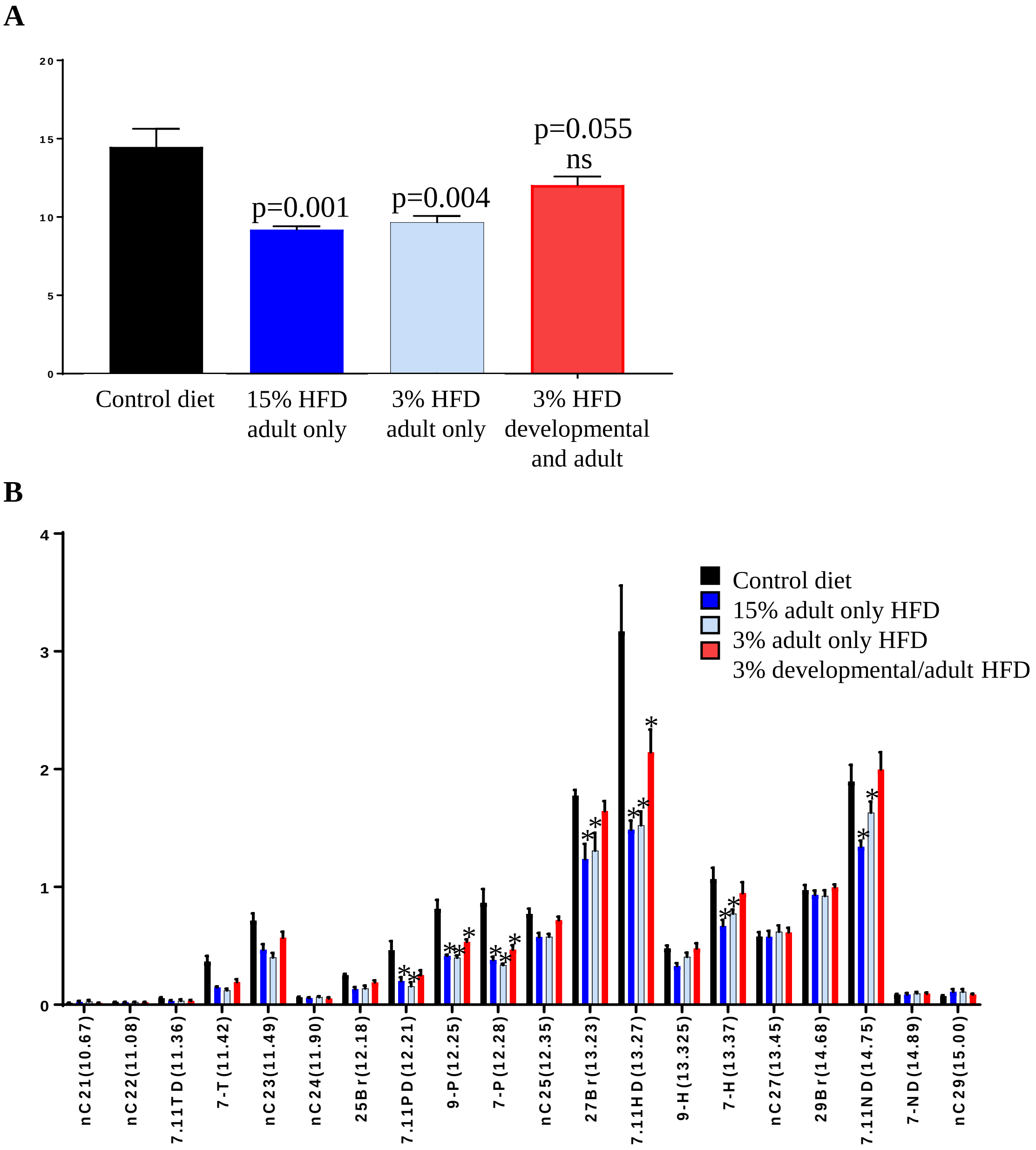

Supplemental Figure 3. Effects of HFD on absolute amounts of CHCs in females. (A) Total CHCs in ng. (B) Absolute amounts of individual CHCs. * represent significant differences in comparison to the control diet.

A

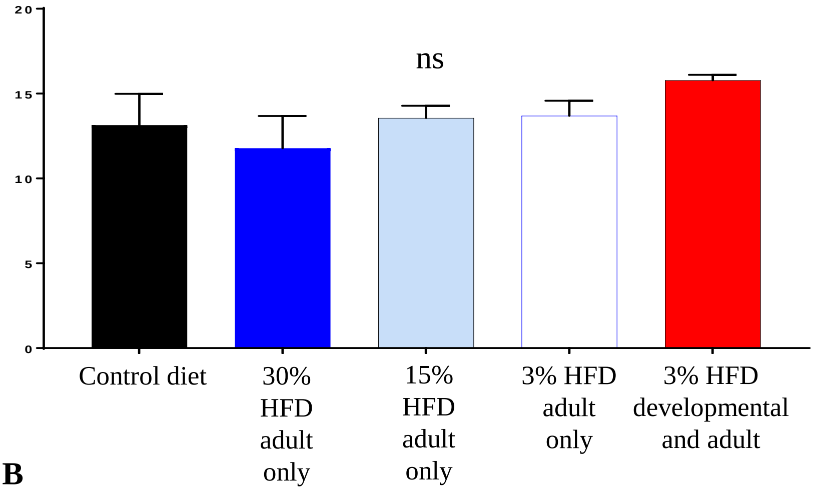

B

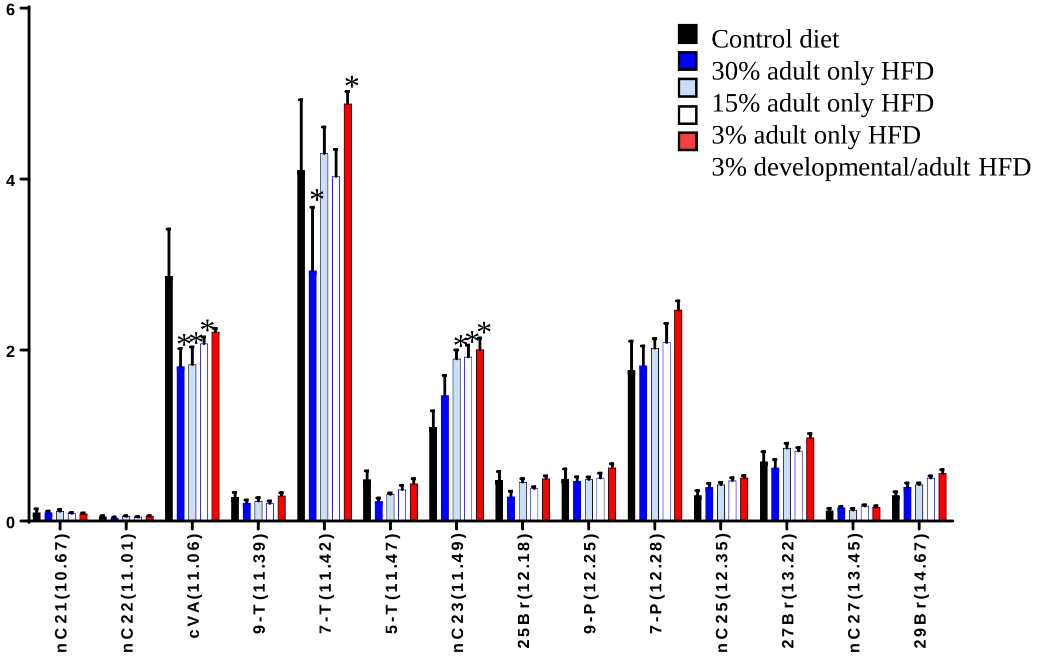

Supplemental Figure 4. Effects of HFD on absolute amounts of CHCs in males. (A) Total CHCs in ng. (B) Absolute amounts of individual CHCs. * represent significant differences in comparison to the control diet.

Supplemental Table 1. ANOVA results for the effects of developmental 3% HFD on male mating behaviors in single-pair mating assays in light and dark conditions with intact females.

Light/Dark	Parameter	Analysis	DF	*F*	*P*
Light	Log Courtship Latency	ANOVA	121	12.9183	**<0.0001**
		Female Diet	1	5.4497	**0.0213**
		Male Diet	1	28.8278	**<0.0001**
		Female*Male	1	4.2460	0.0415
Light	Arcsine Courtship Index	ANOVA	120	4.5272	**0.0048**
		Female Diet	1	3.5332	0.0626
		Male Diet	1	1.5076	0.2220
		Female*Male	1	8.4096	**0.0045**
Dark	Log Courtship Latency	ANOVA	118	5.2346	**0.0020**
		Female Diet	1	11.1145	**0.0012**
		Male Diet	1	0.5827	0.4468
		Female*Male	1	4.3249	0.0398
Dark	Arcsine Courtship Index	ANOVA	117	0.1168	0.9501
		Female Diet	1	0.2350	0.62887
		Male Diet	1	0.0988	0.7539
		Female*Male	1	0.0303	0.8621

Values in bold are statistically significant at Bonferroni corrected α=0.025.

Supplemental Table 2. ANOVA results for the effects of developmental 3% HFD on male mating behaviors in single-pair mating assays in light and dark conditions with decapitated females.

Light/Dark	Parameter	Analysis	DF	*F*	*P*
Light	Log Courtship Latency	ANOVA	115	3.0558	0.0313
		Female Diet	1	4.3642	0.0390
		Male Diet	1	4.6687	0.0328
		Female*Male	1	0.2483	0.6193
Light	Arcsine Courtship Index	ANOVA	124	6.3870	**0.0005**
		Female Diet	1	10.9410	**0.0012**
		Male Diet	1	8.6339	**0.0040**
		Female*Male	1	0.0023	0.9616
Dark	Log Courtship Latency	ANOVA	98	3.8501	**0.0120**
		Female Diet	1	9.7660	**0.0024**
		Male Diet	1	0.0689	0.7935
		Female*Male	1	2.8966	0.0920
Dark	Arcsine Courtship Index	ANOVA	109	3.9099	**0.0108**
		Female Diet	1	9.4545	**0.0027**
		Male Diet	1	1.1998	0.2758
		Female*Male	1	1.9626	0.1642

Values in bold are statistically significant at Bonferroni corrected $\alpha=0.025$.

Supplemental Table 3. ANOVA results for the effects of adult-only 3% HFD on mating behaviors in single-pair mating assays.

Parameter	Analysis	DF	*F*	*P*
Log Courtship Latency	ANOVA	99	0.5191	0.6702
	Female Diet	1	0.0121	0.9126
	Male Diet	1	1.2197	0.2722
	Female*Male	1	0.3253	0.5689
Arcsine Courtship Index	ANOVA	98	2.1527	0.0987
	Female Diet	1	1.6536	0.2016
	Male Diet	1	2.0500	0.1555
	Female*Male	1	2.6995	0.1037
Female Activity	ANOVA	98	5.6555	**0.0013**
	Female Diet	1	15.5924	**0.0002**
	Male Diet	1	0.7931	0.3754
	Female*Male	1	0.7337	0.3938
Male Activity	ANOVA	98	4.5576	**0.0050**
	Female Diet	1	11.3461	**0.0011**
	Male Diet	1	1.7164	0.1933
	Female*Male	1	0.7819	0.3788
Log Mating Latency	ANOVA	98	5.1040	**0.0026**
	Female Diet	1	10.4559	**0.0017**
	Male Diet	1	1.4104	0.2380
	Female*Male	1	3.2885	0.0729

Values in bold are statistically significant at Bonferroni corrected $\alpha=0.01$.

Supplemental Table 4. ANOVA results for the effects of adult-only 7% HFD on mating behaviors in single-pair mating assays.

Parameter	Analysis	DF	*F*	*P*
Log Courtship Latency	ANOVA	96	2.6701	0.0521
	Female Diet	1	1.2524	0.2660
	Male Diet	1	0.1681	0.6827
	Female*Male	1	6.7947	0.0106
Arcsine Courtship Index	ANOVA	96	2.1578	0.0983
	Female Diet	1	3.3466	0.0705
	Male Diet	1	0.0000	0.9957
	Female*Male	1	3.0596	0.0836
Female Activity	ANOVA	99	17.6635	**<0.0001**
	Female Diet	1	48.4933	**<0.0001**
	Male Diet	1	3.4781	0.0652
	Female*Male	1	1.0191	0.3153
Male Activity	ANOVA	99	8.8919	**<0.0001**
	Female Diet	1	24.5406	**<0.0001**
	Male Diet	1	0.4652	0.4968
	Female*Male	1	1.6701	0.1994
Log Mating Latency	ANOVA	98	4.2367	**0.0074**
	Female Diet	1	10.7587	**0.0015**
	Male Diet	1	0.6828	0.4107
	Female*Male	1	1.2283	0.2705

Values in bold are statistically significant at Bonferroni corrected α=0.01.

Supplemental Table 5. ANOVA results for the effects of adult-only 15% HFD on mating behaviors in single-pair mating assays.

Parameter	Analysis	DF	*F*	*P*
Log Courtship Latency	ANOVA	103	4.1775	**0.0079**
	Female Diet	1	4.0863	0.0459
	Male Diet	1	1.1214	0.2922
	Female*Male	1	7.1324	**0.0088**
Arcsine Courtship Index	ANOVA	103	1.1989	0.3142
	Female Diet	1	2.5627	0.1126
	Male Diet	1	0.6905	0.4080
	Female*Male	1	0.2745	0.6015
Female Activity	ANOVA	101	8.9851	**<0.0001**
	Female Diet	1	24.0652	**<0.0001**
	Male Diet	1	1.1635	0.2834
	Female*Male	1	1.3873	0.2417
Male Activity	ANOVA	101	5.2609	**0.0021**
	Female Diet	1	15.2366	**0.0002**
	Male Diet	1	0.0539	0.8169
	Female*Male	1	0.4887	0.4862
Log Mating Latency	ANOVA	98	10.8396	**<0.0001**
	Female Diet	1	31.2037	<**0.0001**
	Male Diet	1	1.8281	0.1796
	Female*Male	1	0.0759	0.7835

Values in bold are statistically significant at Bonferroni corrected α=0.01.

Supplemental Table 6. Results for the effects of adult-only 30% HFD male on mating behaviors in single-pair mating assays.

Parameter	Analysis	DF	*t*	*P*
Log Courtship Latency	t Test	43.5566	0.8904	0.1891
Arcsine Courtship Index	t Test	47.3376	-2.7007	**0.0096**
Female Activity	t Test	51.7931	-3.4085	**0.0013**
Male Activity	t Test	52.0143	-3.7063	**0.0005**
Log Mating Latency	t Test	52.3464	2.9586	**0.0046**

Values in bold are statistically significant at Bonferroni corrected $\alpha=0.01$.
